# Complete Genome Sequence of *Streptomyces* Phage Sentinel

**DOI:** 10.1128/MRA.01339-20

**Published:** 2021-01-07

**Authors:** Andrew Talcott, Russell Moreland, Jolene Ramsey, James Clark, Isla Hernandez, Mei Liu, Ben Burrowes

**Affiliations:** a Department of Biochemistry and Biophysics, Texas A&M University, College Station, Texas, USA; b Center for Phage Technology, Texas A&M University, College Station, Texas, USA; Portland State University

## Abstract

The *Streptomyces* genus produces over two-thirds of clinically useful, natural antibiotics. Here, we describe the isolation and genome annotation of siphophage Sentinel, which utilizes *Streptomyces* sp. strain Mg1 as a host. It has a 50,272-bp genome and 83 protein-coding genes and shows similarity to other *Streptomyces* phages in the *Arequatrovirus* genus.

## ANNOUNCEMENT

*Streptomyces* spp. are Gram-positive, filamentous bacteria typically found in soil (1). They are rarely pathogenic and play an important role in the pharmaceutical industry, as members of this genus produce over two-thirds of naturally occurring antibiotics used clinically (2, 3). The uncharacterized strain *Streptomyces* sp. Mg1 has been shown to be directly antagonistic to Bacillus subtilis through the production of chalcomycin A, a macrolide antibiotic (4). Phage Sentinel is a novel double-stranded DNA (dsDNA) siphophage, which utilizes *Streptomyces* sp. Mg1 as a host.

Sentinel was isolated by plaque purification as described previously (5) from a topsoil sample collected in Houston, Texas, in July 2019, utilizing the host strain *Streptomyces* sp. Mg1 (6) (provided by Paul Straight, Texas A&M University). Culture conditions were 30°C and nutrient broth supplemented with 10 mM MgCl_2_, 8 mM Ca(NO_3_)_2_, and 0.5% glucose. DNA was purified using a Wizard DNA clean-up kit as described previously (7) and prepared as Illumina TruSeq libraries with 300-bp inserts using a Nextra Flex kit. The libraries were sequenced on an Illumina MiSeq platform with paired-end 150-bp reads using V2 (300-cycle) chemistry. The 471,318 reads were visualized with FastQC (www.bioinformatics.babraham.ac.uk/projects/fastqc) and manually trimmed with FastX 0.0.14 (http://hannonlab.cshl.edu/fastx_toolkit/download.html) and then assembled using SPAdes v3.5.0 (8) into a contig with a mean coverage of 324.3×. PCR and Sanger sequencing were used to close the genome with the primers CTCCTCGCGGTCATGTG (forward) and GCATCGTCATCCCGAAGATC (reverse). Protein-coding genes were predicted by GLIMMER v3 (9) and MetaGeneAnnotator v1.0 (10), and detection of tRNAs was by ARAGORN v2.36 (11). Protein function was identified by conserved domain searches with InterProScan v5.33 (12), sequence similarity searches were performed with BLAST v2.9.0 (13), and transmembrane domain identification was done through TMHMM v2.0, all on default settings (14). BLAST v2.9.0 searches (accessed on 23 April 2020) were conducted against NCBI nonredundant, Swiss-Prot, and TrEMBL databases (15). ProgressiveMauve v2.4 was used to determine a genome-wide DNA sequence similarity between Sentinel and other phages (16). All annotation tools were accessed using the Galaxy platform hosted by https://cpt.tamu.edu/galaxy-pub (17–19). Further annotation analysis was conducted with the use of HHpred, which implements HH-suite v3 (20) software. Phage morphology was identified as that of a siphovirus (data not shown) by negatively staining samples with 2%(wt/vol) uranyl acetate and viewed by transmission electron microscopy at the Texas A&M Microscopy and Imaging Center.

The 50,272-bp genome of Sentinel has a G+C content of 66.86%, lower than the host genome G+C content of 72.17% (6). Coding density is 93.62% with a total of 83 protein-coding genes identified. Sentinel was classified as a temperate phage due to the presence of putative integrase and immunity repressor genes (21, 22). Comparative genomics revealed a >60% nucleotide sequence similarity to 10 phages found in the *Arequatrovirus* genus. The highest nucleotide identity observed was a 65.26% similarity to *Streptomyces* phage Diane (GenBank accession number MF766046.1). A putative function was assigned to 36 proteins, with the remaining 47 proteins classified as hypothetical. BLASTp analysis showed 66 putative proteins shared (E value, <0.001) between *Streptomyces* phages SqueakyClean (GenBank accession number MF766047.1) and Sentinel.

### Data availability.

The genome of Sentinel was deposited in GenBank with accession number MT701597.1. The associated BioProject, SRA, and BioSample accession numbers are PRJNA222858, SRR11574901, and SAMN14609633, respectively.
